# Use of antipsychotics and long-term risk of parkinsonism

**DOI:** 10.1007/s10072-021-05650-z

**Published:** 2021-10-15

**Authors:** Angelo d’Errico, Elena Strippoli, Rosario Vasta, Gianluigi Ferrante, Stefania Spila Alegiani, Fulvio Ricceri

**Affiliations:** 1 Epidemiology Unit, Piedmont Region, ASL TO3, Grugliasco, Italy; 2 ALS Center, ‘Rita Levi Montalcini’ Department of Neuroscience, University of Turin, Via Cherasco, 15, 10126 Turin, Italy; 3 National Centre for Drug Research and Evaluation, National Institute of Health (ISS), Rome, Italy; 4 Center for Oncology Prevention Piemonte, Città della Salute e della Scienza, Turin, Italy; 5 Department of Clinical and Biological Sciences, University of Turin, Turin, Italy

**Keywords:** Neuroleptics, Atypical parkinsonisms, Parkinson’s disease, Epidemiology, Drug-induced parkinsonism

## Abstract

**Introduction:**

Few epidemiological studies have assessed the risk of parkinsonisms after prolonged use of neuroleptics. We aimed to examine the long-term risk of degenerative parkinsonisms (DP) associated with previous use of neuroleptics.

**Methods:**

All residents in Piedmont, Northern-west Italy, older than 39 years (2,526,319 subjects), were retrospectively followed up from 2013 to 2017. Exposure to neuroleptics was assessed through the regional archive of drug prescriptions. The development of DP was assessed using the regional archives of both drug prescriptions and hospital admissions. We excluded prevalent DP cases at baseline as well as those occurred in the first 18 months (short-term risk). The risk of DP associated with previous use of neuroleptics was examined through Cox regression, using a matched cohort design.

**Results:**

The risk of DP was compared between 63,356 exposed and 316,779 unexposed subjects. A more than threefold higher risk of DP was observed among subjects exposed to antipsychotics, compared to those unexposed (HR = 3.27, 95% CI 3.00–3.57), and was higher for exposure to atypical than typical antipsychotics. The risk decreased after 2 years from therapy cessation but remained significantly elevated (HR = 2.38, 95% CI 1.76–3.21).

**Conclusions:**

These results indicate a high risk of developing DP long time from the start of use and from the cessation for both typical and atypical neuroleptics, suggesting the need of monitoring treated patients even after long-term use and cessation.

**Supplementary Information:**

The online version contains supplementary material available at 10.1007/s10072-021-05650-z.

## Introduction

Parkinsonisms include a variegate group of diseases characterized by a combination of bradykinesia, tremor at rest, rigidity or loss of postural reflexes, flexed posture, and freezing [1]. Parkinson’s disease (PD) is the most frequent form, followed by atypical parkinsonisms including multiple system atrophy (MSA), dementia with Lewy bodies (DLB), progressive supranuclear palsy (PSP) and corticobasal degeneration (CBD), and other less frequent forms [1]. Secondary parkinsonisms mostly develop as side effect of several drugs, in particular antipsychotics, antidepressants, calcium channel blockers, antiarrhythmics, antihistamines, antiepileptics, and others [2]. The drug-induced parkinsonism (DIP) is the most frequent cause of parkinsonism after PD [3], with a proportion ranging from less than 10% to more than 50% in different studies [2, 4, 5]. DIP was firstly described with “typical” neuroleptics as a consequence of their antagonistic effect on D_2_ dopaminergic receptors, eventually leading to a reduced dopamine neurotransmission [6]. Second-generation “atypical” neuroleptics [7] show a lower antagonistic activity on D_2_ dopaminergic receptors and less likely increase the risk of parkinsonism [8].

According to the literature, DIP generally arises within few months from the drug assumption and ends within 6 months after the drug has been stopped. However, in up to 15–20% of cases, symptoms persist for longer time, even years [9]. Few studies have evaluated the risk of DIP onset with a longer latency [10–13].

Here, we assessed the long-term risk of PD and atypical parkinsonisms after 18 months from the initiation of neuroleptics using a large cohort followed up for several years through hospital admissions and drug prescriptions. A secondary aim was to evaluate whether the risk linked to atypical neuroleptics was lower than that of typical ones, as reported by several studies.

## Methods

### Study population

Data from the Longitudinal Study of Piedmont were used [14]. This is a health monitoring system based on individual record linkage, for all residents in the region (more than 4 million people), between 2011 population census data, mortality registers, hospital admissions (since 2010), archives of drug prescriptions (since 2010), and records of direct ambulatory drugs distribution (since 2012). Subjects aged over 39, participating in the 2011 census, still resident and alive on January 1, 2013, were enrolled in the study cohort (*n* = 2,526,319). Prevalent cases were excluded and identified based on at least two antiparkinsonian drugs prescriptions, including any drug in the Anatomical Therapeutic Chemical (ATC) class N04, or a hospital admission with PD, secondary or atypical parkinsonism as principal or secondary diagnosis (ICD-9 codes for DLB 331.82, PD 332.0, secondary parkinsonism 322.1, CBD 331.6, MSA and PSP 333.0) in the year before the start of the study (2012).

The period of observation started on January 1, 2013, and ended on December 31, 2017, or until the date of death or emigration out of the region.

### Outcome ascertainment

The outcome was defined as the development of PD or atypical parkinsonisms and was identified through both drug prescriptions and records of direct drug ambulatory distribution and hospital admissions during the observation period. PD and atypical parkinsonisms will be referred from now on as degenerative parkinsonisms (DP). In particular, subjects with at least 1 year of therapy (1 year elapsed between the first and last date of prescription) and five medication packages (one prescription may include up to three medication packages) of levodopa or levodopa derivatives (ATC class N04BA) during that period were considered DP cases. As a consequence, drug prescriptions from 2018 were also used to identify DP cases with a first prescription in 2017. The cut-off of at least five medication packages prescribed during 1 year was decided arbitrarily as a compromise between the need to preserve statistical power of the analysis and that of having an outcome characterized by relatively high specificity, given that in our study population, the median of medication packages per year was equal to six. The temporal criterion of at least 1 year between the first and the last prescription was set because a definitive diagnosis, in particular for PD, is based also on patient’s response to levodopa, which may take several months to be assessed.

DP patients were also identified through the principal diagnosis in the hospital discharges data (ICD-9 codes: 331.82, 332.0, 331.6, 333.0). Outcome onset was set as the date of the first levodopa prescription or of the first hospital admission occurring during follow-up.

### Exposure ascertainment

Exposure to antipsychotics was assessed between 2012 and 2017 using the drug prescriptions and ambulatory distribution archives, defining subjects as exposed if they had at least two prescriptions of any antipsychotic drug (ATC class N05A). Based on the ATC code, neuroleptics were classified into three groups: typical antipsychotics (including phenothiazines, butyrophenons, thioxanthenes, piperidine, and diphenylbutylpiperidines); atypical antipsychotics (including indole derivatives, substituted benzamides, dibenzodiazepines, benzisoxazole derivatives, quinolones, aripripazole, and others); and lithium derivatives.

### Statistical analysis

**Fig. 1:**
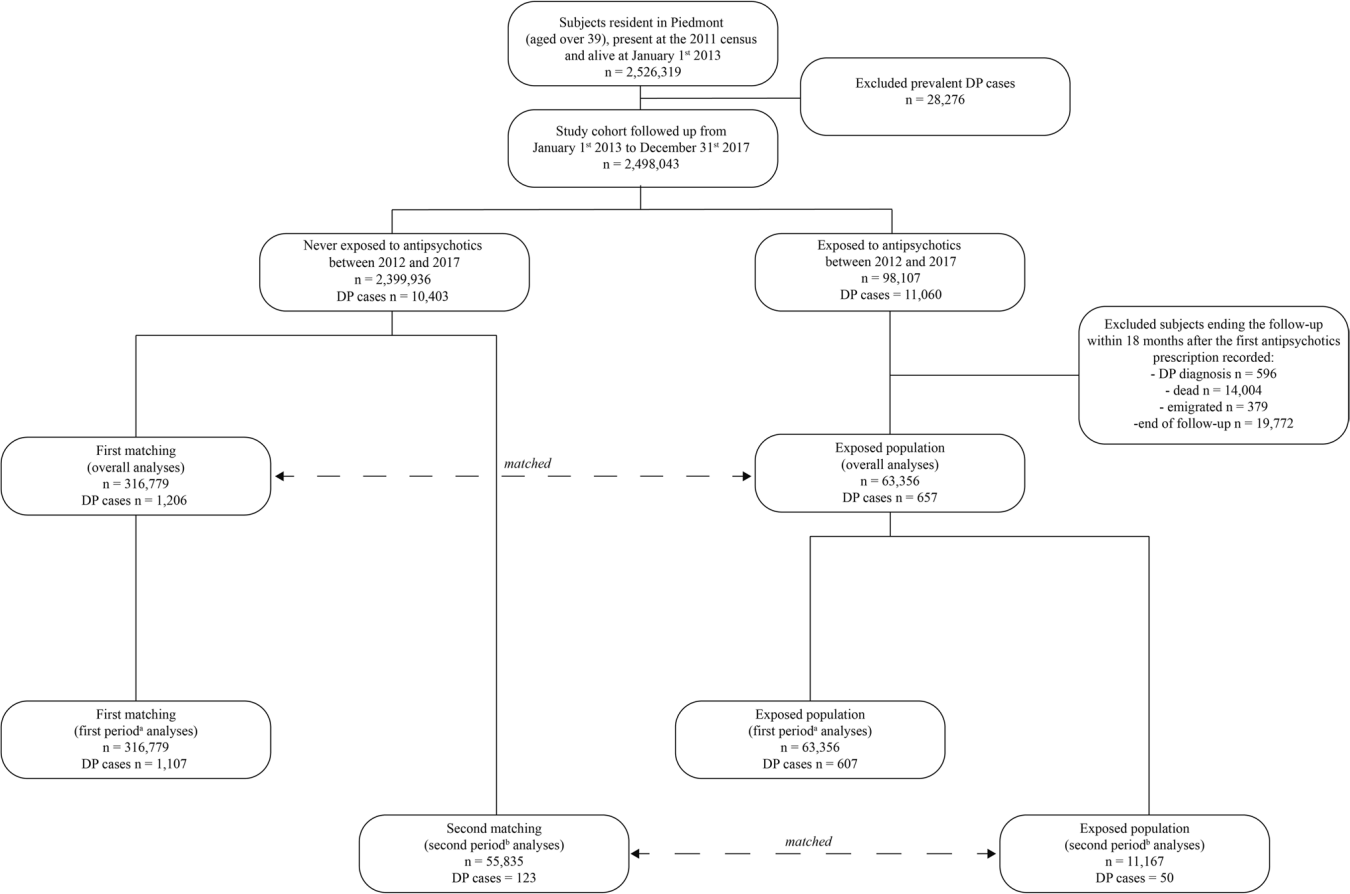
Flowchart of the selection of the study population

The study was conducted using a matched cohort design: each exposed subject was randomly matched to five unexposed subjects within strata defined by sex, age at census (5-year classes), and date of start of follow-up (date of matching). A first matching was performed by setting the date of matching for the exposed subjects at 18 months after the first antipsychotics prescription, as the objective of the study was to assess the long-term DP risk associated with antipsychotics exposure (Fig. 1). Subjects who developed a parkinsonism or ended the follow-up within 18 months after the first antipsychotics exposure were excluded from the analyses (*n* = 34,751). The matching process eliminated basic demographic differences between groups and allowed dealing with the immortal time bias [15].

We also aimed to assess whether DP risk was associated with antipsychotics exposure after drug withdrawal. This analysis was performed by dividing the follow-up into two sub-periods (Fig. 1s). The first one ended after 2 years from therapy cessation, while the second sub-period started 2 years from antipsychotics withdrawal and ended on December 31, 2017, or until the date of DP occurrence, death, or emigration out of the region. For the analysis on the second sub-period, we did a new matching between the remaining formerly exposed subjects after 2 years from drug cessation (who were still alive and have not developed the outcome) and a new group of not exposed to antipsychotics (five unexposed per one exposed subject) by sex, age at census in 5-year classes, and date of start of follow-up (Fig. 1; Figure 1S).

We performed time to event analyses, estimating hazard ratios (HRs) of developing DP associated with exposure to antipsychotics through Cox proportional hazard regression models, following the methodology previously described for the analysis of matched cohort data [16, 17].

Exposure to antipsychotics was also examined by antipsychotics typology (typical, atypical, typical + atypical, lithium). For this purpose, a dataset with type of antipsychotics as a time-varying variable was constructed in order to account for different therapy combinations over time, considering each individual as exposed to typical, atypical, or lithium from the date of first prescription, per type, to the end of follow-up. Because of the small number of subjects treated with lithium, the association between DP and exposure to this drug was not evaluated for the combination with other types of antipsychotics, although it was also treated as a time-varying variable in the analyses.

Differences in HRs of DP between users of typical and atypical antipsychotics were tested for statistical significance (*p* < 0.05) assessing heterogeneity of the HRs through random effect meta-analysis, using the “metan” Stata command. All analyses were performed using Stata, version 13.

In addition, a sensitivity analysis restricted to subjects exposed to antipsychotics, also treated as time-varying variables, was performed to assess the risk of DP by antipsychotics typology, using typical neuroleptics as the reference category.

Analyses were adjusted for sex, age (in 10-year classes, treated as a time-varying variable), and socioeconomic characteristics available at census. After preliminary analyses conducted comparing Akaike information criterion (AIC) and Bayesian information criterion (BIC) indexes among bivariate models (exposure plus each socioeconomic variable), we included in the final model educational level (university degree; high school diploma; middle school and vocational school diploma; elementary school diploma; or no formal education), household type (couple with children; couple without children; single parents or single member household; subjects living in cohabitation or in institutions), and employment status (employed; retired; other condition). The proportional hazard assumption in the models for overall exposure to antipsychotics and for antipsychotic type was also checked.

Statistical analyses were performed using the STATA software (13^th^ version).

## Results

A total of 2,463,292 subjects were followed up. In the overall analysis, we compared 63,356 subjects exposed to antipsychotics with 316,779 unexposed subjects. The same set of individuals was used in the analyses on DP risk within 2 years from therapy cessation, but truncating the follow-up of unexposed subjects when the matched exposed subjects ended their follow-up (in correspondence of outcome onset or after 2 years from drug cessation), while in the analyses on DP risk after 2 years from therapy’s end, 11,167 subjects exposed were matched to 55,835 unexposed individuals (Table 1).

**Table 1:** Distribution of number and percentage of subjects exposed and not exposed to antipsychotics by demographics, socioeconomics characteristics, and outcome

	Exposed overall		Unexposed				Exposed in the second period		Unexposed	
			Original cohort		First matching				Second matching	
	*N*	(%)	*N*	(%)	*N*	(%)	*N*	(%)	*N*	(%)
Subjects	63,356		2,399,936		316,779		11,167		55,835	
DP diagnosis										
Yes	657	(1.04)	10,403	(0.43)	1,206	(0.38)	50	(0.45)	123	(0.22)
No	62,699	(98.96)	2,389,533	(99.57)	315,573	(99.62)	11,117	(99.55)	55,712	(99.78)
Sex										
Male	23,768	(37.51)	1,124,948	(46.87)	118,839	(37.51)	4,009	(35.90)	20,045	(35.90)
Female	39,588	(62.49)	1,274,988	(53.13)	197,940	(62.49)	7,158	(64.10)	35,790	(64.10)
Age classes										
< 50	11,566	(18.26)	673,074	(28.05)	57,830	(18.26)	1,955	(17.51)	9,775	(17.51)
50–59	10,046	(15.86)	568,824	(23.70)	50,230	(15.86)	1,756	(15.72)	8,780	(15.72)
60–69	10,189	(16.08)	513,277	(21.39)	50,945	(16.08)	1,963	(17.58)	9,815	(17.58)
70–79	14,537	(22.94)	420,805	(17.53)	72,685	(22.95)	2,678	(23.98)	13,390	(23.98)
≥ 80	17,018	(26.86)	223,956	(9.33)	85,089	(26.86)	2,815	(25.21)	14,075	(25.21)
Educational level										
Degree	3,251	(5.13)	209,120	(8.71)	21,255	(6.71)	631	(5.65)	3,624	(6.49)
High school	8,703	(13.74)	500,023	(20.83)	51,304	(16.20)	1,628	(14.58)	8,989	(16.10)
Middle school and vocational school	20,853	(32.91)	961,379	(40.06)	105,284	(33.24)	3,670	(32.86)	18,584	(33.28)
Elementary school or no formal education	30,549	(48.22)	729,414	(30.39)	138,936	(43.86)	5,238	(46.91)	24,638	(44.13)
Household type										
Couple with children	12,238	(19.32)	885,150	(36.88)	84,210	(26.58)	2,276	(20.38)	14,807	(26.52)
Couple without children	18,190	(28.71)	726,481	(30.27)	101,090	(31.91)	3,282	(29.39)	18,001	(32.24)
Single parents	6,333	(10.00)	194,681	(8.11)	24,269	(7.66)	997	(8.93)	4,405	(7.89)
Single member	22,589	(35.65)	539,729	(22.49)	99,213	(31.32)	3,948	(35.35)	17,237	(30.87)
Cohabitations or in institution	4,006	(6.32)	53,895	(2.25)	7,997	(2.52)	664	(5.95)	1,385	(2.48)
Occupational condition										
Employed	10,864	(17.15)	1,045,903	(43.58)	92,348	(29.15)	2,323	(20.80)	15,968	(28.60)
Retired	37,405	(59.04)	966,052	(40.25)	176,212	(55.63)	6,471	(57.95)	31,303	(56.06)
Other condition	15,087	(23.81)	387,981	(16.17)	48,219	(15.22)	2,373	(21.25)	8,564	(15.34)
Other causes of follow-up end										
Emigration	1,096	(1.73)	55,279	(2.30)	5,048	(1.59)	215	(1.93)	570	(1.02)
Death	18,767	(29.62)	239,046	(9.96)	47,610	(15.03)	2,683	(24.03)	6,277	(11.24)
Censored	42,836	(67.61)	2,095,208	(87.30)	262,915	(83.00)	8,219	(73.60)	48,865	(87.52)

After matching, the groups studied had the same distribution by sex and age but still remained some relatively small differences about household type, employment status, and education, which were all statistically significant (*p* < 0.001), due to the large size of the cohort (Table 1). During the follow-up period, 11,060 incident DP cases were identified in the cohort (Table 1, 4^th^ column), 10,198 (92%) through drug prescriptions, and 861 (8%) through hospital admissions. The latter included 595 PD, 190 MSA or PSP, and 76 DLB cases. Among the 11,060 DP cases, 657 (5.9%) had been exposed to antipsychotics. In the first matching group, mean follow-up duration was 3.08 years (SD = 1.88) for subjects exposed to antipsychotics and 3.42 years (SD = 1.82) for unexposed subjects, while in the second matching group (after 2 years from neuroleptics cessation), mean duration was 1.76 years (SD = 1.29) for exposed subjects and 1.94 years (SD = 1.32) for the unexposed. Among subjects exposed to antipsychotics, 607 developed DP during therapy or within 2 years from the end of the therapy, while 50 had DP after 2 years from therapy’s end (Table 2). In the overall analysis, most exposed subjects had therapies composed of atypical (56.8%) or a mixture of typical and atypical (24.7%) antipsychotics, while in the second sub-period, starting after 2 years from therapy’s end, therapies including only typical (23.5%) or atypical (60.2%) antipsychotics prevailed. Lithium was used by a small proportion of exposed subjects (8.1%), higher in the first, compared to the second sub-period (14.1% and 5.1%, respectively).

**Table 2:** Hazard ratios (HR) of degenerative parkinsonisms (DP) associated with exposure to antipsychotics and type of drug, overall and by sup-periods of follow-up (before and after 2 years from therapy cessation), in the matched cohort (matched by sex, age, and sub-period). Cox regression models adjusted for educational level, household type, and employment status

	Overall			First period^a^		Second period^b^		
	*N* (%)	DP cases	HR (95% CI)	DP cases	HR (95% CI)	*N* (%)	DP cases	HR (95% CI)
Unexposed	316,779 (83.3)	1,206	1	1,107	1	55,835 (83.3)	123	1
Exposed of which (% exposed):	63,356 (16.7)	657	3.27 (3.00–3.57)	607	3.33 (3.04–3.64)	11,167 (16.7)	50	2.38 (1.76–3.21)
Typical or atypical								
Typical	9,934 (15.7)	87	1.99 (1.59–2.49)	71	1.86 (1.45–2.37)	2,619 (23.5)	16	2.33 (1.36–3.98)
Atypical	35,991 (56.8)	412	3.47 (3.10–3.89)	381	3.56 (3.16–4.00)	6,720 (60.2)	31	2.50 (1.70–3.68)
Typical and atypical	15,626 (24.7)	130	2.90 (2.39–3.51)	128	2.98 (2.45–3.61)	1,519 (13.6)	2	1.17 (0.27–5.13)
Lithium^c^	5,266 (8.3)	88	3.82 (2.86–5.10)	86	3.73 (2.79–5.00)	566 (5.1)	2	6.11 (0.85–44.14)

^a^During therapy, starting from 18 months after therapy start and ending after 2 years from antipsychotics withdrawal

^b^After 2 years from antipsychotics withdrawal

^c^Lithium exposure was not treated in combination with other antipsychotics types

During the whole observation period, a more than threefold DP higher risk was observed among subjects exposed to antipsychotics, compared to those unexposed (HR = 3.27, 95% CI 3.00–3.57). The risk in the first sub-period was similar to that in the overall follow-up (HR = 3.33, 95% CI 3.04–3.64), as 90% of exposed cases were concentrated here, and decreased by about one-third after 2 years from antipsychotics cessation (HR = 2.38, 95% CI 1.76–3.21).

The HR of DP associated with exposure limited to typical antipsychotics (HR = 1.99, 95% CI 1.59–2.49) was significantly lower than that for exposure only to atypical antipsychotics (HR = 3.47, 95% CI: 3.10–3.89) (heterogeneity test: *p* < 0.001), considering the whole observation period, with exposure to mixed therapies of typical and atypical neuroleptics showing a risk intermediate between the two (HR = 2.90, 95% CI 2.39–3.51). Differences in risk between typical and atypical antipsychotics disappeared after 2 years from antipsychotics cessation, when both groups showed HRs around 2.5, although both estimated on a limited number of exposed cases (16 and 31, respectively) (Table 2). Lithium had the highest HR of DP in the first period (HR = 3.72, 95% CI 2.77–4.98), whereas the results for lithium and for mixed neuroleptics in the second sub-period were based on too few cases to be meaningfully interpreted (only 2 cases each).

Results regarding neuroleptics typology were substantially confirmed in the sensitivity analysis restricted to the exposed population (Table 3), which showed a 50% higher risk for patients treated with only atypical (HR = 1.50, 95% CI 1.21–1.86) or with typical + atypical therapy (HR = 1.48, 95% CI 1.14–1.91), compared to those treated only with typical neuroleptics. The highest HR of DP among the exposed was again estimated for lithium, with a two-fold risk (HR = 2.06, 95% CI 1.60–2.65), compared to the therapy with only typical neuroleptics; proportional hazard assumption was violated for exposure to lithium, although with a low correlation between Schoenfeld residuals and time.

**Table 3:** Hazard ratios (HR) of degenerative parkinsonisms (DP) for exposure to antipsychotics by drug typology, restricting to exposed subjects. Cox regression models adjusted for sex, age, education level, household type, and employment status

	Only exposed analysis				
		*N*	(%)	DP cases	HR (95% CI)
Subjects exposed		63,356	(100)	657	
Typical or atypical					
	Typical	9,934	(15.7)	87	1
	Atypical	35,991	(56.8)	416	1.50 (1.21–1.86)
	Typical and atypical	15,626	(24.7)	134	1.48 (1.14–1.91)
Lithium^a,b^		5,266	(8.3)	88	2.06 (1.60–2.65)

^a^Lithium exposure was not treated in combination with other antipsychotics types

^b^Violation of proportional hazard assumption (rho = 0.10561, *p*-value = 0.0029)

## Discussion

The present study showed an increased risk of developing DP long time after starting an antipsychotics therapy. The risk was more than three times higher, compared to unexposed subjects, in the sub-period beginning after 18 months from start of use until 2 years after cessation, and decreased by about one-third, although remaining significantly elevated, after 2 years from cessation of these drugs. The observed threefold risk of DP for exposure to antipsychotics corresponds to an attributable proportion to the exposure of 67%, indicating that, among those treated, two-thirds of DP cases would have been caused by the drugs.

These results suggest that parkinsonisms arising after the use of antipsychotics are not necessarily transient and could develop even long after drug cessation.

Among the few available cohort studies, a risk of parkinsonism almost two-fold higher was found among antipsychotics users, compared to non-users, in an elderly population in the USA [18], while a Canadian study estimated a relative risk almost 70% higher among users [19]. However, both studies had a short follow-up and were not able to assess whether a higher risk of parkinsonism persisted with longer therapy duration or after drug withdrawal. In another longitudinal study, in which psychiatric patients treated with neuroleptics for more than 1 year were evaluated for movement disorders during a 4-year follow-up (but with a mean follow-up of only 1.1 year), more than half cases showed persistent symptoms of parkinsonism at follow-up, indicating that such symptoms have frequently long duration [20]. Furthermore, a Korean case–control study estimated a three-fold higher risk of parkinsonism associated with current use of antipsychotics [21].

To our knowledge, only case reports and case series have been published on the long-term risk of parkinsonism linked to neuroleptics [10–12], whereas no epidemiological studies have evaluated this association over periods of time longer than 1 year, except for a 15-year French prospective study. Consistently with our results, this study showed an increased risk of incident PD by more than three times following exposure to antipsychotics, after excluding subjects who developed extrapyramidal symptoms during drug use [13]. It is noteworthy that in this study, only 35% of the parkinsonism cases occurred while under treatment with these drugs, which would support long-term neurological effects of neuroleptics.

DIP and DP, especially Parkinson’s disease, are not easily distinguishable. Many characteristics could be useful in the differential diagnosis, namely symptoms symmetry, the presence of tremor at rest, and non-motor symptoms [22], although all these features could be seen in both forms. Some ancillary investigations, such as transcranial sonography of the substantia nigra, the Dopamine transporter single-photon emission tomography (SPECT DaT SCAN), and the 123I-metaiodobenzylguanidine (MIBG) cardiac scintigraphy, could be helpful. However, further studies assessing their accuracy in clinical practice are needed, and the differential diagnosis is still challenging [22] For example, in a Spanish survey on more than 5,000 subjects, all the 26 DIP cases found in the study were newly diagnosed cases, which had not been identified previously [23]. Furthermore, diagnosis of DIP is normally made according to strict temporal criteria (starting of symptoms within 6 months from drug initiation and reversibility of symptoms within 6 months after drug cessation) [5, 23, 24], which may limit the identification of DIP cases and lead to an underestimation of their prevalence. Especially if occurring after long-term exposure or after exposure cessation, it seems that DIP may be misclassified as PD [6], apparently not only by general practitioners and psychiatrists, but also by neurologists [25, 26].

Our results suggest that a substantial part of DP cases treated with neuroleptics are actually tardive forms of DIP passed unrecognized or misdiagnosed, possibly because of their late onset, or a positive response to dopa, or the resemblance of their clinical symptomatology with PD or other forms of DP. The observation of a high risk of DP also after long time from its cessation would also indicate that in some cases, the damage caused by neuroleptics to the nigrostriatal dopaminergic function may be irreversible [27]. However, it has been proposed that DIP cases persisting for such a long time after the offending drug has been withdrawn may be pre-symptomatic PD cases, in which neuroleptic therapy has unmasked a pre-existing PD and anticipated its onset [6, 24], as suggested by the presence at autopsy of pathological findings typical of PD in patients who recovered from DIP after drug cessation [28, 29].

Surprisingly, DP risk resulted higher with atypical antipsychotics, despite their lower antagonistic activity on D_2_ dopaminergic receptors, compared to typical ones [6]. This issue is still controversial and is likely complicated by the use of drugs at different dosage and with different potency in the various studies. A meta-analysis of clinical trials comparing typical and atypical antipsychotics found a higher risk of extrapyramidal symptoms for exposure to typical drugs but mainly limited to use of these drugs at high dosage, whereas when chlorpromazine at low doses (< 600 mg) was compared to atypical agents, the incidence of extrapyramidal symptoms was similar [30]. Moreover, the risk of extrapyramidal effects seems to vary within atypical antipsychotics, with clozapine showing significantly lower extrapyramidal effects than other common atypical neuroleptics [31]. Finally, only typical agents with higher potency, such as haloperidol, perphenazine, or thiothixene, were found to pose a higher risk than atypical ones [19]. Further studies focused at assessing the risk of developing DP after the use of atypical antipsychotics could help in disentangling this unexpected finding.

Although our results could have been biased by the possibility that patients treated with antipsychotics could have received higher surveillance for the development of parkinsonian symptoms, the strong difference between typical and atypical neuroleptics risks suggests that the association with DP was attributable to use of these medications.

A high risk of DP was found also for exposure to lithium. A previous Danish study showed that patients treated with lithium had an 80% increased risk of purchasing antiparkinsonian drugs [32]. In a Canadian study, the incidence of dopaminergic drug use almost doubled among subjects treated only with lithium monotherapy, compared with antidepressant monotherapy [33]. However, since lithium could be used to treat mood disorder, its association with parkinsonism could be due to reverse causality and being the effect of a prodromic PD [34]. Nonetheless, these results suggest that also treatment with lithium may involve an increased risk of parkinsonism, hypothesis which should be examined in future studies.

Main strengths of this study are the large population investigated and the rather long follow-up, which gave the possibility of assessing long-term effects of use of neuroleptics, as well as to examine the risk of DP by type of drug. Also, the advanced statistical analysis performed, in which exposure to antipsychotics was treated as a time-varying variable, allowed to estimate precisely time-to-event in relation to the beginning of the exposure. Furthermore, the use of administrative data prevented the possibility of differential misclassification of the exposure and of the outcome. Finally, DP cases in this study represented all cases of corresponding age originated from the Piedmont population during the observation period, while the matched sample was randomly selected from the whole resident population in the region, which makes unlikely that it was affected by some sort of selection.

We are aware of some limitations of this study. Although analyses were adjusted for education and employment status, we did not take into account other possible confounders such as exposure to other drugs, pesticides, metals, organic solvents [35], physical inactivity, tobacco smoking, and alcohol drinking [36].

Reverse causality due to treatment with neuroleptics of psychotics symptoms associated with DP should be also considered. However, it appears unlikely that this bias could have affected our results, since symptoms so severe to be treated with antipsychotics mainly occur in later phases of PD and other forms of parkinsonisms [37]. Contrarily, we considered only DP incident cases after the exclusion of prevalent cases through hospital admissions for DP and use of any antiparkinsonian drug in the year before start of follow-up.

While the use of administrative data for the assessment of the outcome and of the exposure is protective against differential misclassification, it probably introduced some degree of non-differential misclassification of exposure and/or the outcome in the study. However, hospital admissions and drug prescriptions of antiparkinsonian medications for identifying PD have shown to guarantee high positive predictive values (PPV) in identifying PD, in particular for prescription of levodopa [38–41]. As more than 90% of DP cases in our study were identified through prescriptions of levodopa, it is likely that our case series included only a minor proportion of neurological syndromes different from DP. Furthermore, the criteria for case ascertainment based on drugs were quite stringent, as they consisted of having at least five levodopa or dopa-derivatives prescriptions for a duration longer than 1 year. Since DIP generally does not respond to levodopa and its derivatives, it seems difficult that more than a few DIP cases could have been treated for 1 year with these drugs, as they presumably would not have benefited from their effects and would have quitted them. For example, in a US study, only twelve out of more than 100 patients affected by DIP had been treated with levodopa, and only two of them showed some response to treatment [42].

In conclusion, we found a high risk of DP associated with long-term use of neuroleptics, which persisted after 2 years from their cessation. It is still controversial if tardive parkinsonian symptoms should be interpreted as tardive DIP or as the unmasking of a pre-existing PD. However, our findings should raise the awareness about the high risk of extrapyramidal disorders linked to the use of antipsychotics, both typical and atypical, and on the necessity of monitoring patients even after long-term use of these drugs, as well as after their cessation.

## Supplementary Information

Below is the link to the electronic supplementary material.

Supplementary file1 (PNG 18 KB) Figure 1S. Subgroups of exposed subjects according to drug start and cessation.
