# Supplementary figures and images for: Use of antipsychotics and long-term risk of parkinsonism
Source: Neurol Sci. 2021 Oct 15;43(4):2545–53. doi: 10.1007/s10072-021-05650-z (PMC8918175; doi:10.1007/s10072-021-05650-z)

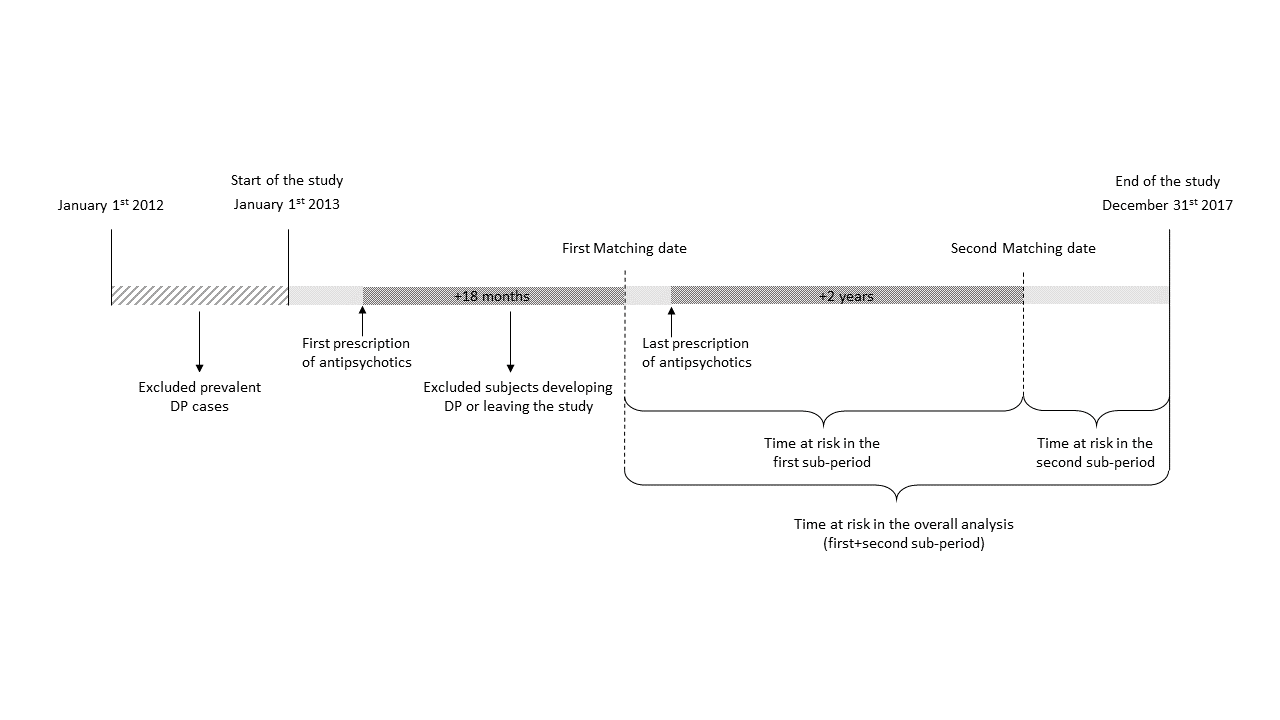

Supplement: Supplementary file 1 — Supplementary file1 (PNG 18 KB) Figure 1S. Subgroups of exposed subjects according to drug start and cessation. [file 10072_2021_5650_MOESM1_ESM.png]
